# Surgery for primary malignant tumors of the abdominal wall: experiences of three African surgical oncology units and review of the literature

**DOI:** 10.1186/s12957-023-03125-3

**Published:** 2023-07-31

**Authors:** Nayi Zongo, Adeline R. Djiguemde, Paratyandé Bonaventure Yameogo, Sidy Ka, Bangaly Traoré, Ahmadou Dem

**Affiliations:** 1 Digestive and General Surgery Unit, Yalgado Ouedraogo Teaching Hospital of Ouagadougou, Joseph Ki-Zerbo University of Ouagadougou, 03 BP 7021 Ouagadougou, Burkina Faso; 2 Joliot Curie Institute of Dakar (Senegal)Cheikh Anta Diop University of DakarCheikh Anta Diop University, 10700 Dakar, Senegal; 3 Surgical Oncology Unit, Donka Hospital of Conakry, Gamal Abdel Nasser University of Conakry, Conakry, Guinea

**Keywords:** Lumpectomy, Parietal repair, Abdominal mesh, Skin flaps

## Abstract

**Background and objectives:**

Tumors of the abdominal wall are uncommon but diverse. The surgical challenge is double. The tumor must be completely removed and the abdominal wall repaired. Our aim was to describe the indications, techniques, and results of surgery on these tumors in an African context.

**Methods:**

Retrospective, multicentric and descriptive study conducted in three West African surgical oncology units. We included all abdominal wall tumors followed up between January 2010 and October 2022. Histological type, size, surgical procedure, and method of abdominal wall repair were considered. Survival was calculated using the Kaplan–Meier method and comparisons of proportions were made using the Student *t* test.

**Results:**

We registered 62 tumors of the abdominal wall and we operated on 41 (66.1%). The mean size of the tumors was 14.3 ± 26 cm. Dermatofibrosarcoma and desmoid tumor were present in 33 and 3 cases respectively. In 31.7% of cases in addition to the tumour, the resections carried away the muscular aponeurotic plane. Parietal resections required the use of a two-sided prosthesis in 6 cases. In 13 cases, we used skin flaps. The resections margins were invaded in 5 cases and revision surgery was performed in all of them. Incisional hernia was noticed in 2 cases. The tumor recurrence rate was 12.2% with an average time of 13 months until occurrence. Overall survival at 3 years was 80%.

**Conclusions:**

Surgery is the mainstay of treatment for abdominal wall tumors. It must combine tumor resections and parietal repair. Cancer surgeons need to be trained in abdominal wall repair.

## Introduction

Abdominal wall tumors are uncommon but diverse [1–4]. They can be locally malignant or frankly invasive with a tendency to metastasize [2–5]. The surgical challenge is double: not only must the tumor be treated, but a solid abdominal wall must also be left in place, ideally during the same operation [1, 6, 7]. The surgical procedure depends on the tumor’s size, topography, histological type, but mostly the layers of the abdominal wall involved [6–8]. Resections of malignant tumors involving only the skin and sub-skin can be conceived without aponeurotic resection [6, 7]. On the other hand, malignant tumors invading the muscular aponeurotic plane require deep resections taking out the muscles and the aponeurosis [6, 7]. In all cases, surgery must repair the abdominal wall at the same time or later, to allow the patient have a normal life [8–10]. Several parietal repair techniques are described in the literature, including the use of a bifacial prosthesis to avoid intestinal complications [1, 11, 12]. The use of adjuvant treatments such as chemotherapy, targeted therapies, or radiotherapy depends on the histological type and the stage of the tumor [10, 13, 14]. The evolution depends on the histological type and the quality of the resections [15–17]. Besides tumor recurrences, parietal complications such as incisional hernias are noted in the literature [9, 18–20]. In the African context, few studies have focused specifically on tumors of the abdominal wall. Furthermore, they are not specifically devoted to surgical aspects. That is why, we undertook this study which reports the experience of three West African surgical oncology units, to better explain the indications, techniques and results of surgery for primary malignant tumors of the abdominal wall in a context of limited resources.

## Patients and methods

### Type and period of study

This is a cross-sectional survey conducted between 1st January, 2010 and 30th October, 2022. It included all patients treated for a primary malignant tumor of the abdominal wall.

### Study site

The study took place in two West African countries with limited resources: Burkina Faso and Guinea. In these countries, less than 5% of the population has health insurance. Consultations occur late and the management of tumors must take into account not only the stage of the disease, but also the financial means of patients and their families. Data was collected in three surgical oncology units: two in Ouagadougou and one in Conakry. In Ouagadougou, the General and Digestive Surgery Departments of the Yalgado Ouedraogo Teaching Hospital and the Protestant Medical Center Schiphra served as our collection framework. In Conakry, the Surgical Oncology Unit of Donka Hospital served as our study framework. These units are standard health structures for the management of digestive, skin, and soft tissue tumors in these two countries.

### Study population

We were interested in all patients followed for a malignant tumor of the abdominal wall in these three structures during the study period. We exhaustively included all patients with usable clinical file. The available data should be enough to assess the surgical procedure.

### Data collection procedures

We collected the data using a questionnaire. Our data sources were the clinical records of patients, the registers of consultation and operative reports. The patients included had their information recorded in the reception registers when they were admitted to the units. In addition, the surgeons who operate on the patients always produce a surgical report which constitutes a source of data. During their hospitalization, the information is put in their file. Once their wounds had healed, they were seen again on the thirtieth postoperative day, and then every 3 months by their surgeon. This information is also put in their files. For each patient, we were interested in the socio-demographic data (age, sex, place of residence), histological type, indications for surgery, surgical procedures performed, adjuvant treatments, carcinological and parietal complications and survival data.

### Data management and analysis strategy

After data collection, we proceeded with data entry using a microcomputer equipped with KoboCollect software version 1.30.1. Excel 2010 and R version 4.1.0 software were used for analysis. We divided the tumors into three groups based on size. We distinguished between small tumors (< 5 cm), medium-sized tumors (5–10 cm), and large tumors (> 10 cm). We then assessed the procedures performed according to the size, the histological type of the tumor and whether or not the muscular aponeurotic plane was affected. The comparisons between the types of resections according to the histological type, the state of damage or not of the muscular aponeurotic plane, the excision margins, the evolution after the surgery and the abdominal complications were possible thanks to the Student's t-test. Survival was assessed using the Kaplan–Meier method.

### Ethical aspects

The study was authorized by the managements of the hospitals and the heads of the surgical units concerned. Data was collected anonymously and confidentiality was respected for all patients.

## Results

### General data

Over 12 years and 10 months, we noted 62 cases of primitive malignant tumors of the abdominal wall. There were 38 women (61.3%), i.e., a sex ratio of 0.6. The mean age of the patients was 39 ± 17.3 years with extremes of 23 and 68 years. Patients had a WHO performance status below II in 93.5% of cases on admission.

**Fig. 1:**
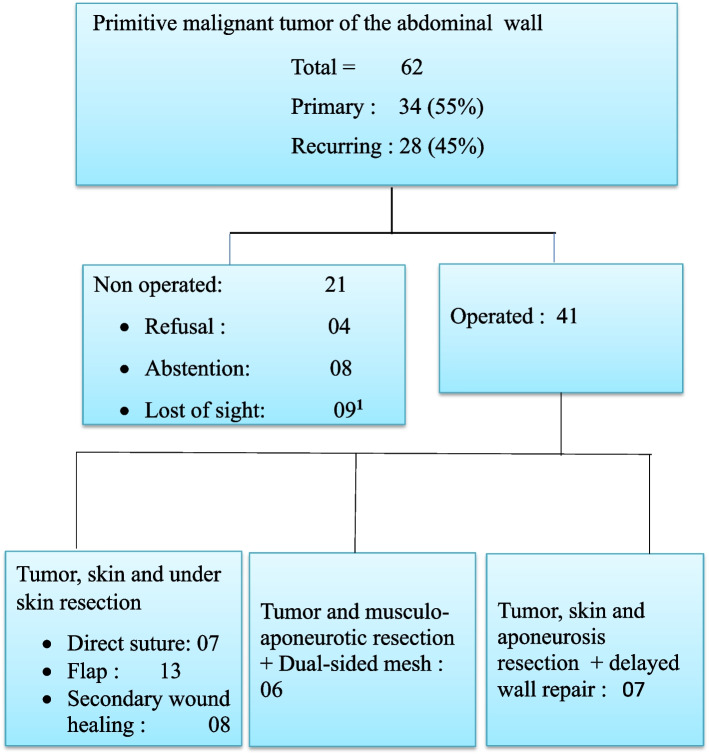
Flow chart. 1: Patients lost from sight before starting any treatment

**Fig. 2:**
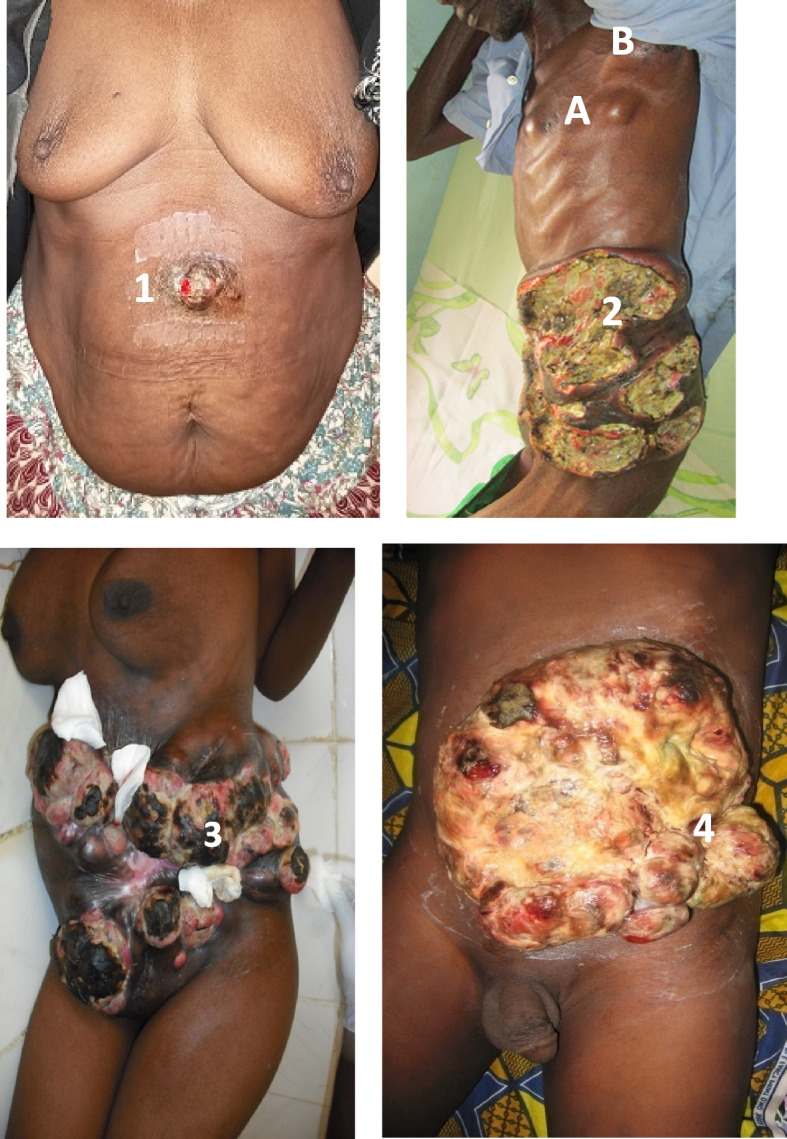
Patients with abdominal wall tumors not operated for various reasons. 1 Dermatofibrosarcoma of the abdominal wall not operated on, due to the patient’s refusal. 2 Lymphoma of the anterolateral abdominal wall in a 52-year-old HIV + patient treated with chemotherapy. Appearance of thoracic nodules (**A**) and axillary lymph nodes (**B**). 3 Multiple recurrence dermatofibrosarcoma taking ¾ of the anterolateral wall. 4 Ulcero-necrotic-granulating and metastatic liposarcoma of the abdominal wall in a 38-year-old patient

The tumors were primary in 28 cases (44.2%) and recurrent in 34 cases (54.8%) (Fig. 1). The reasons for consultation were swelling of the abdominal wall (83.9%), abdominal pain (40.3%), and bleeding (8%). Physical examination revealed a nodular, granulating, necrotic, or hemorrhagic anterolateral abdominal wall mass in 91.9% (Fig. 1). They were attached to the abdominal wall, not very mobile in 16.1% of cases and mobile appearing superficial in 83.9% of cases. The average tumor size was 14.3 ± 26 cm with extremes of 2.5 and 41 cm (Fig. 2, Table 1). Histologically, it was Darier and Ferrand dermatofibrosarcoma in 77.4%, fibrosarcoma in 4.8%, desmoid tumor in 6.4%, liposarcoma in 6.4%, leiomyosarcoma in 1.6%, Schawnonne tumor in 1.6% and neurofibrosarcoma in 1.6%. We noted fixation to the pelvic bones in 4.8% and grazing of the external genitalia in 3.2% of cases. The tumors were metastatic to the lungs (11.3%) and liver (6.6%) at the time of diagnosis.

**Table 1:** Distribution of operated patients according to tumor size, surgical procedure, excision margins, and evolution *n* = 41

Surgical procedure	Tumor, skin and under skin resections	Tumor and musculo-aponeurotic resections	Tumor, skin and aponeurosis resections	Total
Characteristics				
Histologic type				
Dermatofibrosarcoma	26	01	06	33
Desmoïd tumor	00	03	00	03
Liposarcoma	01	01	01	03
Leiomyosarcoma	00	01	00	01
Schawnnoma	01	00	00	01
Tumor size				
< 2 cm	01	01	00	02
< 5 cm	06	01	00	07
> 5 cm	13	01	02	16
> 10 cm	08	03	05	16
State of excision margins				
R0	25	06	05	36
R1	03	00	02	05
Type of skin repair				
Direct skin suture	07	00	00	07
Skin flap	13	00	00	13
Secondary wound healing	08	00	00	08
Meshs	00	06	07	13
Evolution after surgery				
Local recurrence	04	00	01	05
No local recurrence	18	05	04	27
Metastasis	02	00	01	03
Lost from sight	04	01	01	06
Abdominal wall complication				
Incisional hernia	00	00	02	02
Evisceration	00	00	00	00
Suppuration	02	00	02	04
No complication	26	06	03	35
Total	28	06	07	41

*R0* macroscopically and microscopically healthy margins, *R1* macroscopically invaded

### Surgery for tumors of the abdominal wall

We opted for surgical abstention in 8 cases including 4 cases due to metastasis and 3 cases due to non-resectability. Non-resectability was related to tumors of the anterolateral abdominal wall involving the pelvic bones and/or the iliac and femoral vessels. We also renounce the resections of a large full-thickness wall tumor in a patient without the option of a two-sided prosthesis (Fig. 2). Such the resections would expose the patient to a high risk of infection or even serious sepsis. Refusal of surgery despite its technical and financial feasibility was noted in 4 cases (Figs. 1 and 2). Nine patients were lost to follow-up before treatment had started. Surgery was performed in 41 cases (66.1%). Lateral and deep resections margins varied between 3 and 5 cm. The dual purpose of the surgery was taken into account. It involved combining oncological resection and repair of the abdominal wall. To better describe the surgical gestures, we divided the patients into 3 groups.

#### Group 1

**Fig. 3:**
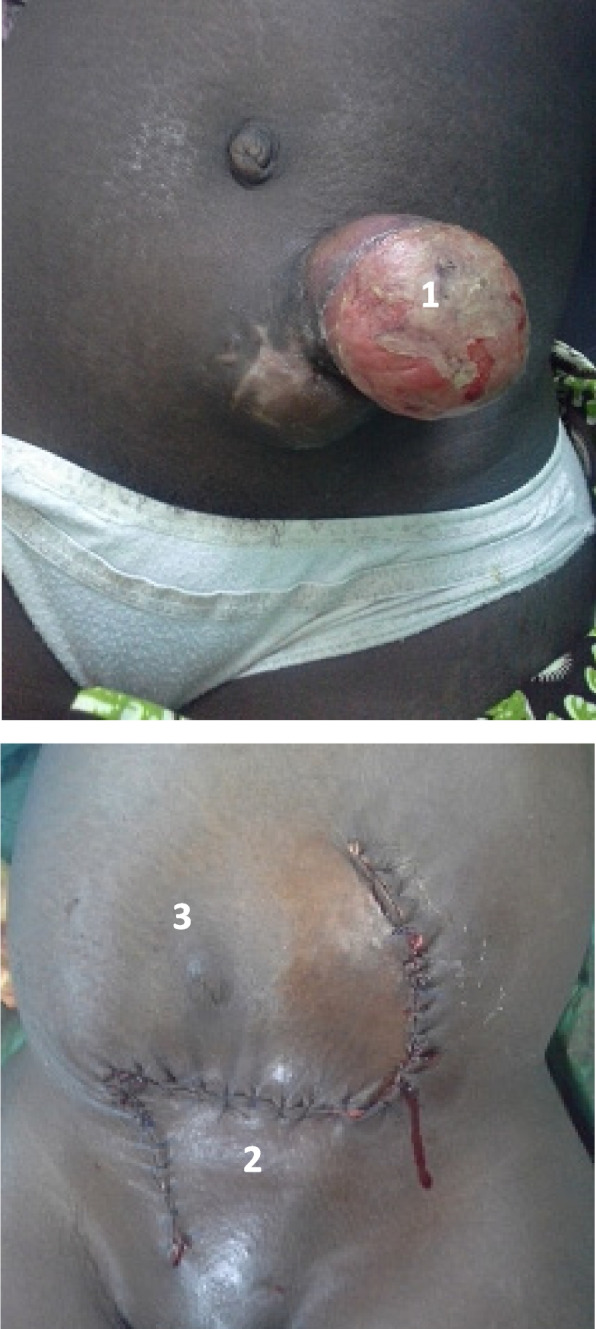
Dermatosarcoma of the abdominal wall recurrent in a 6-month pregnancy treated by resection and covering skin flaps. **1** Ulcerated and necrotic recurrent tumor. **2** Closure with skin flaps. **3** Pregnancy

**Fig. 4:**
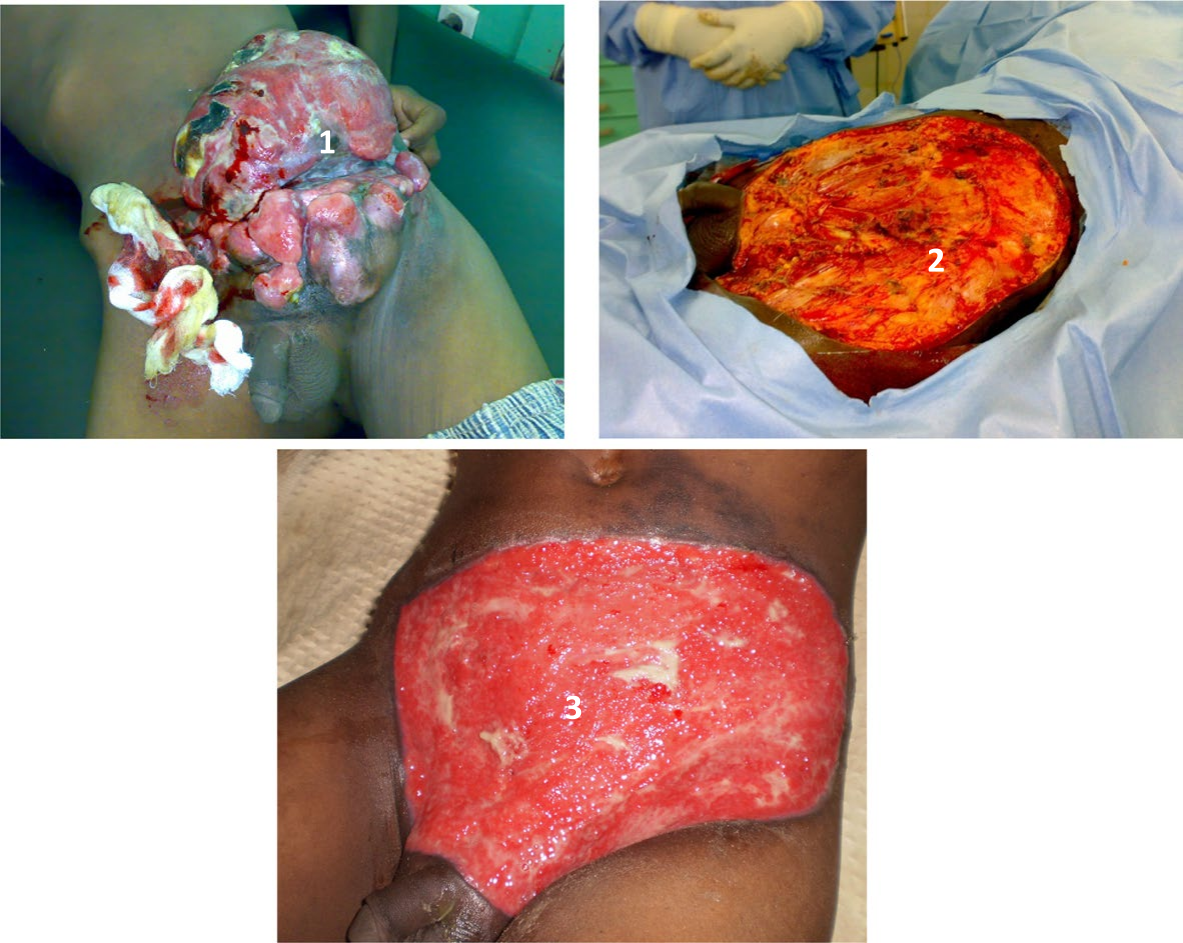
Dermatofibrosarcoma of the anterior abdominal wall in an 18-year-old patient at his 2nd iterative. **1** Ulcero-necrotic, granulating, and hemorrhagic tumor. **2** Operative wound. **3** Second intention wound healing option, filling after more than 2 months of dressing

It concerned tumors involving only the skin and sub-skin, superficial, mobile, i.e., 68.3% of cases. We performed a lateral and deep in sano resections without removing the aponeurosis (Table 1). In sano resections means complete resection without macroscopic or microscopic residue. This parietal repair consisted of direct skin suture in 7 cases (17.1%). Large tumors (> 10 cm), exuberant very superficial, pendular, involving only the dermis and the epidermis, were treated with the same procedures by means of mobilization of skin flaps in 13 cases, i.e., 31.7% of the operated cases. The O to Z plasty technique was used in 9 out of 13 cases (Fig. 3). Controlled wound healing was the option in 8 cases (19.5%) after the resections of a large tumor leaving significant defects in place without the possibility of direct closure or mobilization of flaps (Table 1, Fig. 4).

#### Group 2

**Fig. 5:**
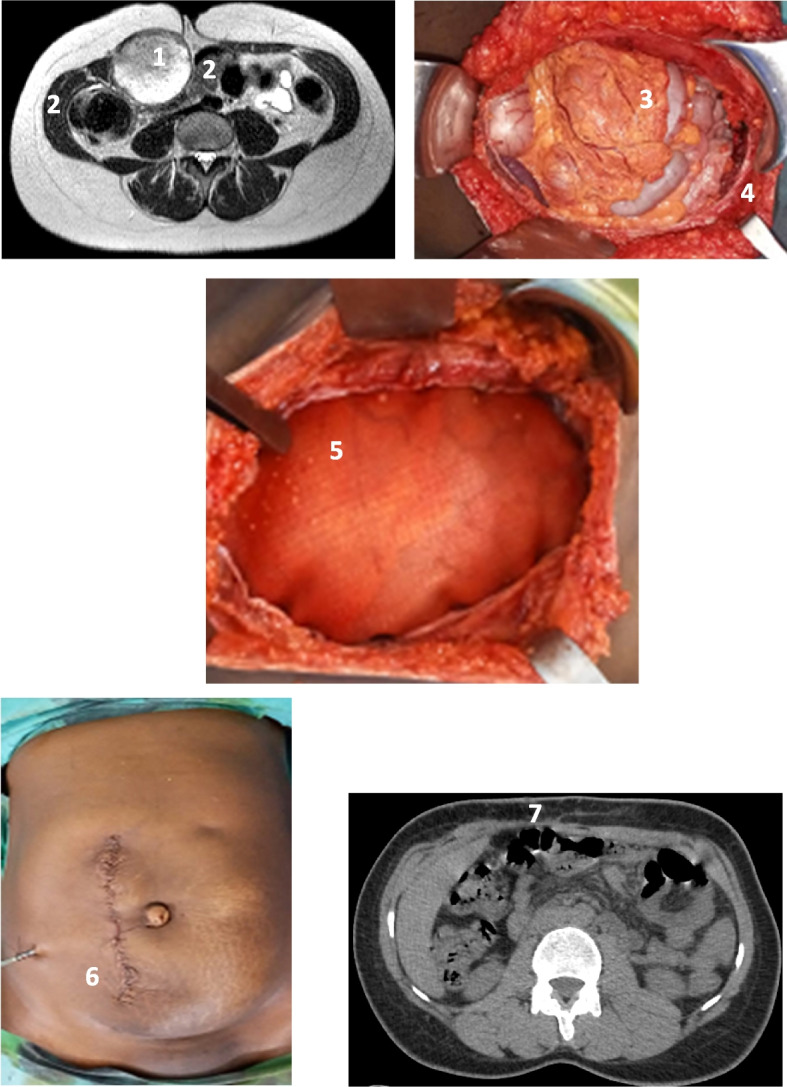
Desmoid tumor of the abdominal wall embedded in the rectus abdominis muscle. 1 Intramuscular abdominal wall tumor. 2 Muscle. 3 Appearance after muscular aponeurotic resection. 4 Edge of the aponeurosis. 5 Placement of a bifacial mesh. 6 Skin closure and appearance at the end of the procedure. 7 Abdominal mesh in place 3 months after surgery

The second group of tumors consisted of those presenting muscular aponeurotic invasion of the abdominal wall (31.7%). For these cases, we carried wide and complete resections, taking up the entire thickness of the abdominal wall in 2 cases. The skin was spared in 4 cases. The surgery then left a significant parietal defect without the possibility of direct suturing for the aponeurosis (Fig. 5). We then implanted a two-sided prosthesis. The bifacial mesh, i.e., a two-sided prosthesis is an abdominal prosthesis with a smooth side that can be in contact with the viscera (intestines) without creating adhesions, and a second, non-smooth, adherent side that will be in contact with the components of the abdominal wall. The edges of the prosthesis were anchored to the edges of the aponeurosis by separate stitches of 2/0 non-absorbable monofilament (Fig. 7). The size of the prosthesis was determined by the diameter of the parietal defect. The parietal repair was completed by covering the skin (Fig. 5).

#### Group 3

In a small group of patients admitted with necrotic masses (17.1%), we performed skin, sub-skin, tumor and superficial layer of the aponeurosis resections in one piece. Dressings and antibiotic therapy were applied. The parietal repair was performed later (Fig. 5).

In summary, monobloc tumor resections with the skin and the sub-skin were performed in front of the dermatofibrosarcomas in 26/33 cases (*p* < 0.0001). Skin flaps were used after resections which removed the skin and sub-skin in 11/16 cases of tumors of 5 to 10 cm (*p* = 0.02). Controlled wound healing was the option for tumors larger than 10 cm (*p* < 0.0001). Tumor and muscular aponeurotic resections were performed in 3/3 cases for desmoid tumors and 3/38 for all other histological types (*p* < 0.0001). Prostheses were used after the resections removing the aponeurosis or the muscular aponeurotic plane in 13/13 cases (*p* = 0.0001).

### Adjuvant treatments

Radiotherapy is not available in our two countries. Patients who can afford the cost of travel and radiotherapy go to neighboring countries. External radiotherapy was associated in 3 cases, i.e., 7.3% of patients operated. Targeted therapy, i.e., imatinib mysilate, was used as an adjuvant therapy in 7 cases (21.2%) of Darier and Ferrand dermatofibrosarcomas. It was a large, necrotic tumor with a high mitotic index. The average duration of treatment was 12 months at a dose of 400 mg per day. Adjuvant chemotherapy was used in 2/3 cases of sarcomas. It was palliative in front of the appearance of metastases in 7 cases.

### Evolution

Extemporaneous anatomopathological examination was not obtained in any case. Histological results were obtained within 3 weeks after surgery. The margins were invaded in 5 cases (12.2%). Revision surgery was performed in all these cases. The parietal complication rate such as suppuration and incisional hernia was 14.6% and 4.9% respectively. In four out of six cases the suppurations were noticed during the resections taking away the superficial layer of the aponeurosis carried out in front of necrotic tumors followed by deferred parietal repair (Table 1).

The tumor recurrence rate was 12.2% with an average time of occurrence of 13 months (Table 1). In 4 cases out of 5, the recurrences concerned dermatofibrosarcomas of Darier and Ferrand (Table 1) and were not observed in any particular type whether or not the aponeurosis was involved (*p* = 0.92). Abdominal wall strength was completely restored in 95.1% of cases. The abdominal wall regained normal functionality (protective role of viscera, mobility) in 85.4% of cases. Patients were satisfied with the aesthetic appearance of their abdominal wall in 58.5% of cases. They were very satisfied in 26.8% of cases. They were not satisfied with the presence of unsightly scars in 14.6% of cases. Intermittent residual pain treated with level 1 analgesics was noticed in 7.3% of cases. We observed the appearance of metastases in 4.9% of these operated patients. Overall survival at 5 years was 28/35 (80%).

## Discussion

Primary malignant tumors of the abdominal wall are all malignant tumors which develop at the level of the anterolateral abdominal wall, that is, those located in the parietal region bordered above by the costal edges (margins), below by the inguinal ligaments and laterally by the posterior axillary lines [7, 8]. They are uncommon [2–4]. Dermatofibrosarcomas are the most common, accounting for 77.4% of primary malignant tumors of the abdominal wall in our series. However, all histological types can be found, in particular liposarcomas, rabdomysosarcomas, lymphomas, and desmoid tumors [1–4, 21]. The therapeutic modalities are as diverse as the histological types and the stages at diagnosis [10, 13, 22]. There is a clear contrast between early diagnosis with small tumors in the West and late diagnosis with large sizes in black Africa [17, 23, 24]. Although surgery is the main curative treatment, it is not always feasible [17, 23]. In our series, we sometimes opted to refrain from surgery, because of the unresectability of the tumor and/or the patient’s inability to afford the exorbitant cost of a two-sided prosthesis for parietal repair. In fact, it is not only a matter of performing oncological surgery, but also of ensuring repair after lumpectomy, allowing the viscera to be covered by a solid wall [7, 9, 22, 25]. Abdominal wall tumor surgery is a major surgery involving multiple specialties [7]. Successful reconstruction of the abdominal wall requires a prior multidisciplinary consultation between surgical oncologists, medical oncologists and reconstructive surgeons [6]. In our African context, these teams are difficult to bring together because certain specialties do not exist yet. This is why oncological surgery, parietal reconstruction and drug management are often left to the sole responsibility of the surgical oncologist who has strong background in oncoplastic surgery and medical oncology. All the patients in this series were attended to, operated, and followed up by surgical oncologists in the three surgical oncology units concerned. Several types of surgical procedures were described. In the literature, two types of resections are particularly common [26–28]. First, there is wide lumpectomy removing the tumor, the skin and the sub-skin [6, 7]. The parietal repair is then done by a direct cutaneous suture, a controlled wound healing, or a cutaneous covering thanks to the mobilization of flaps, pedunculated or not [7, 12]. In our series, it was mainly about the mobilization of non-pedicled flaps allowing O to Z oncoplasty. Dermatofibrosarcomas represented 80.5% of the tumors in our series. These tumors tend to adhere and to ulcerate the skin, leaving the muscular aponeurotic plane free. They may therefore allow the muscular aponeurotic plane to be spared during their resections [6, 7]. However, desmoid tumors and sarcomas tend to involve the muscular aponeurotic plane and most often require muscular aponeurotic resections [2, 29, 30]. That is why, the second most common resections method described in the literature consists of muscular aponeurotic resections concomitantly with lumpectomy [11, 14, 19]. This is in line with the concept of Khansa et al. who distinguish three parietal defects after oncological surgery for primary abdominal wall tumors [6]. Type 1 defects involve the skin and subcutaneous tissue only. Type 2 defects involve muscle and fascia only. Type 3 defects involve at the same time the skin, subcutaneous tissue, muscle and fascia [6]. Most type I defects require primary suturing; type II or III defects usually require good repair using flaps and/or prosthesis [6, 7, 21]. In addition to the structures involved in defining the three levels of parietal defects, Anderson proposes to consider the topography of the tumor by dividing the abdominal wall into 4 zones corresponding to different anatomical realities involved in the reconstruction [7]. In some cases, we performed a single-piece resection removing the tumor, the skin, and the sub-skin, as well as the muscular aponeurotic plane, leaving the intra-abdominal viscera bare. Simple polypropylene prosthesis is not suitable after muscular aponeurotic resections, because of the risk of adhesions of the intestines to the prosthesis with consequent occlusions and digestive fistulas. This requires parietal repair with the use of a two-sided prosthesis. Applying the smooth side of the mesh to the intestines prevents their adhesion. Instead of the use of a two-sided prosthesis, the large omentum was used in Egypt after extensive resections of abdominal wall tumor [1]. A pedicle of the greater omentum is anchored to the edges of the muscular aponeurotic resections. This separates the simple polypropylene prosthesis that will be fixed in front of the greater omentum. This prosthesis which is much less expensive than a two-sided prosthesis, will be in contact with the greater omentum and not with the intestines [1]. In our African countries with limited resources, this is a good alternative, because the two-sided prosthesis that we use cost four times more than the simple polypropylene prosthesis used in combination with the omentum.

Other abdominal wall reconstruction methods can be used. Muscle flap mobilizations are possible for abdominal wall reconstruction. Some authors admit that release of the transverse abdominis muscle is an acceptable method in the treatment of large defects and hernias of the abdominal wall, associated with low perioperative morbidity and low recurrence rates [31, 32]. They are good alternatives, especially in our context of limited resources where not all patients have access to mesh.

In our African context of work, the large size of the tumors due to late diagnosis, and sometimes the presence of necrosis, explains why in addition to the surgical methods most commonly used in the literature, we also used other procedures. We performed tumor resections removing the skin and sub-skin, as well as the aponeurosis without closing the skin or repairing the wall immediately. This was related to a significant risk of infection given the necrosis of the tumor. Concomitant prosthesis repair and mobilization of skin flaps are not recommended because of the high risk of infection. In fact, the use of synthetic mesh can lead to complications in infected areas. An alternative not used in our series would be the use of biological mesh such as Permacol® biological mesh [33]. The use of Permacol® surgical implants for abdominal wall repair is safe. The presence of contaminated fields does not seem to influence hernia recurrence when Permacol® biological mesh is used [33]. It would be a good alternative for immediate reconstruction of the abdominal wall, even after the resections of necrotic tumors.

We note that all the surgical procedures adopted for primary malignant tumors of the abdominal wall share with all oncological surgeries the need for resections with healthy margins (R0 resections) [2, 27, 34–36]. It is undoubtedly the parietal repair with its two imperatives of covering the viscera to avoid infections and leaving a solid wall in place to avoid eviscerations and incisional hernias that make surgery for tumors of the abdominal wall unique [2, 27, 34, 35]. In addition, the assessment of evolution after surgical treatment must take into account oncological complications such as recurrences and metastatic evolution, but also parietal complications such as suppuration, evisceration and incisional hernia [9, 13, 18–20, 28, 37]. In our series, we noted 12.2% recurrence. These were mainly Darier and Ferrand dermatofibrosarcomas that had already recurred before being admitted to our centers. This tumor is also known for its high tendency of recurrence [5, 15–17]. In our countries, their large sizes at diagnosis increase their risk of recurrence. Fibrous tumors also have a tendency to recur [38]. However, in our series, we did not note any recurrence, despite the absence of radiotherapy. This was attributed to a wide resection with healthy margins. The resections in sano is the best guarantee of evolution without recurrence of desmoid fibrous tumors although adjuvant treatments also minimize the risk of recurrence [38].

In our series, recurrence was associated with histological type in a very highly statistically significant way (*p* < 0.0001). There was no statistically significant difference in the risk of recurrence between the types of resections removing or not removing the aponeurosis (*p* = 0.92). Whatever the histological type and the characteristics of the tumor, in our series, all the resections were either in sano, and in the contrary case, a revision surgery was made after obtaining the histological results of the operative part. Many other authors also had the same option [17, 23]. The presence of extemporaneous in some countries allows surgeons to perform these resections during the same operation [5, 16]. In the three centers we used for our study framework, frozen section examination is not available. For this reason, surgical revision is done as soon as the histology is obtained. Survival was statistically and significantly better for dermatofibrosarcomas of Darier and Ferrand than for other tumors. This was in line with data from the literature. In fact, dermatofibrosarcoma of Darrier and Ferrand has a predominantly local evolution giving rise to metastases occurring in less than 5% of cases [5, 17]. Survival is rarely involved. In dermatofibrosarcomas, survival and recurrence-free survival are influenced by the use of targeted therapies [39]. Patient survival has improved with the identification of genetic mutations such as translocation *t*(17–22) and the use of mysilate imatinib as neoadjuvant therapy in locally advanced, ulcerated and multiply recurrent tumors [40]. Targeted therapy was used as an adjuvant therapy in 21.2% of Darier and Ferrand dermatofibrosarcomas in our study.

We did not use abdominal wall physiotherapy in our series. However, prior to major abdominal surgery, total body rehabilitation including structured exercise, nutritional optimization, psychological support, and cessation of negative health behaviors, reduces complication rates and improves functional outcomes [41, 42]. Our patients should benefit from this before and after surgery.

### The weaknesses of the study

The study certainly had limitations. The collection was retrospective in countries without a computerized data management system. In addition, the lack of equitable access to diagnosis and quality care explains why some patients die in villages without coming to our care centers. Also, some people go to hospitals, but cannot even get a diagnosis, not talking of treatment, due to a lack of financial resources. There is no universal health insurance in our countries. This is why a large number of patient records did not have sufficient information to be included. The exclusion of these records was a data gap. In addition, the surgical procedures and their results do not seem to be a perfect reflection of all the West African centers where these tumors were operated. In fact, the patients included in this series were operated by cancer surgeons with extensive experience in oncological surgery and abdominal parietal surgery. A prospective study, including a larger number of African centers is needed to better assess the results of the surgical management of these parietal tumors.

## Conclusions

Surgery is the mainstay of treatment for tumors of the abdominal wall. It must combine tumor resections and parietal repair. The tumor resections must be wide and deep in our countries, even without the possibility of extemporaneous histological examination. The extent of the resections is influenced by the size, topography, histological type of the tumor, extension or not to the muscular aponeurotic plane, but also the experience of the surgeon. The use of two-sided prosthesis is necessary for the coverage of the viscera and the solidity of the abdominal wall in lumpectomies with a major muscular aponeurotic resections. Resections in sano reduces the risk of recurrence. Postoperative follow-up, in addition to tumor-related complications, must also consider the possibility of non-tumoral parietal complications such as incisional hernias. An early diagnosis would make this surgery less mutilating with better results. Training of surgeons in the principles of cancer surgery and abdominal parietal surgery, multidisciplinary care involving oncologists, surgeons and plastic surgeons will improve results.

## Data Availability

The data sets used and/or analyzed during the current study are available from the corresponding author on reasonable request.

## References

[1] Abouzid A, Shetiwy M, Hossam A, Abd EM (2022). Abdominal wall reconstruction using omental flap with mesh repair following resection of aggressive abdominal wall neoplasms. Oncol Res Treat.

[2] Wong SL (2008). Diagnosis and management of desmoid tumors and fibrosarcoma. J Surg Oncol.

[3] Bozas G, Anagnostou D, Tassidou A, Moulopoulos LA, Bamias A, Dimopoulos MA (2006). Extranodal non-Hodgkin's lymphoma presenting as an abdominal wall mass. A case report and review of the literature. Leuk Lymphoma.

[4] Khorgami Z, Nasiri S, Rezakhanlu F, Sodagari N (2009). Malignant schwannoma of anterior abdominal wall: report of a case. J Clin Med Res.

[5] Jing C, Zhang H, Zhang X, Yu S (2021). Dermatofibrosarcoma protuberans:a clinicopathologic and therapeutic analysis of 254 cases at a single institution. Dermatol Surg.

[6] Khansa I, Janis JE (2015). Modern reconstructive techniques for abdominal wall defects after oncologic resection. J Surg Oncol.

[7] Mericli AF, Baumann DP, Butler CE (2018). Reconstruction of the abdominal wall after oncologic resection: defect classification and management strategies. Plast Reconstr Surg.

[8] Stojadinovic A, Hoos A, Karpoff HM, Leung DH, Antonescu CR, Brennan MF, Lewis JJ (2001). Soft tissue tumors of the abdominal wall: analysis of disease patterns and treatment. Arch Surg.

[9] Wilkinson MJ, Chan KE, Hayes AJ, Strauss DC (2014). Surgical outcomes following resection for sporadic abdominal wall fibromatosis. Ann Surg Oncol.

[10] Giordano S, Garvey PB, Baumann DP, Liu J, Butler CE (2017). Prior radiotherapy does not affect abdominal wall reconstruction outcomes: Evidence from Propensity Score Analysis. Ann Surg Oncol.

[11] Oudot C, Orbach D, Minard-Colin V, Michon J, Mary P, Glorion C (2012). Desmoid fibromatosis in pediatric patients: management based on a retrospective analysis of 59 patients and a review of the literature. Sarcoma.

[12] Zongo N, Ouedraogo NLM, Windsouri M, Yameogo LSC, Kouchika Chabi TR, Niamba P (2022). Skin oncoplasties: O-to-Z technique a technique of choice in situation of limited resources? Case of Burkina Faso. World J Surg Oncol.

[13] Zhao X, Cao Z, Nie Y, Liu J, Yuan X, Chen J (2021). Retrospective analysis of defect reconstruction after abdominal wall tumor resection in 30 patients. Hernia.

[14] Lahlou MK, Soufi M, Bensaid M, Messrouri R, Benamr S, Essadel H (2009). Fibromatose aggressive de la paroi abdominale (tumeurs desmoïdes). Afr J Cancer.

[15] Kasse A, Dieng M, Deme, A, Fall MC, Drabo B, Timbely G and al. Les dermatofibrosarcomes de darier et ferrand, à propos de 22 caset revue de la littérature. Médecine d’Afrique Noire, 1999, 46 (4), 222–27.

[16] Llombart B, Serra C, Requena C (2018). Sarcomas cutàneo: guidelines for the diagnosis and treatment of cutaneous sarcomas: dermatofibrosarcoma protuberans. Proceedings Dermosiphilogr..

[17] Zongo N, Guigemde RA, Yameogo PB, Somé RO, Traore B, Dem A (2022). Dermatofibrosarcoma protuberans surgery: experiences of four African surgical oncology units and literature review. J Surg Oncol..

[18] Lin SJ, Butler CE (2010). Subtotal thigh flap and bioprosthetic mesh reconstruction for large, composite abdominal wall defects. Plast Reconstr Surg.

[19] Wanjeri JK, Opeya CJO (2011). A massive abdominal wall desmoid tumor occurring in a laparotomy scar: A case report. World J Surg Oncol.

[20] Toyoda Y, Endo W, Kojima F, Matsunaga H, Shimizu K, Yoshioka A, Fujie Y, Fukunaga H, Hojo S, Yoshioka S, Ota H, Terada H, Maeura Y. [A case of liposarcoma of the abdominal wall complicated by thrombocytpenia as a paraneoplastic syndrome ]. 2012 Nov; 39(12):2420–2. Japanese . PMID: 23268097 . 23268097

[21] Song Z, Yang D, Yang J, Nie X, Wu J, Song H, Gu Y (2018). Abdominal wall reconstruction following resection of large abdominal aggressive neoplasms using tensor fascia lata flap with or without mesh reinforcement. Hernia.

[22] Butler CE, Langstein HN, Kronowitz SJ (2005). Pelvic, abdominal, and chest wall reconstruction with AlloDerm in patients at increased risk for mesh-related complications. Plast Reconstr Surg.

[23] Traore B, Zongo N, Diallo TM, Dem A, Sanou A, Diallo AT, and al. Aspects anatomocliniques et thérapeutiques des tumeurs malignes primitives de la paroi abdominale dans deux services de chirurgie de l’Afrique de l’Ouest. Revue Africaine de Chirurgie et Spécialités, 2014 8 (1), 39–45.

[24] Chukwuanukwu TO, Anyanwu SN (2009). Giant fibrosarcoma prostuberans of abodominal wall: management problems in resource-constrained country. Niger J Clin Pract.

[25] Couturaud B, Arnaud E, Revol M, Servant JM (1999). Tumeurs malignes de la paroi abdominale. Dix ans d’expérience à l’Hôpital Saint-Louis. Ann Chir Plast Esthet.

[26] Koshariya M, Shukla S, Khan Z, Vikas V, Pratap Singh A, Baghel P (2013). Giant desmoid tumor of the anterior abdominal wall in a young female: a case report. Case Rep Surg.

[27] Allaw MK, Almola EMA, Rajab WK (2009). Dermatofibrosarcoma protuberans in the anterior abdominal wall: A case report. Tikrit Med J.

[28] Doran H (2012). Recurrent Giant Sarcoma of the Anterior Abdominal Wall. Doran J J Cancer Sci Ther.

[29] Lasser P, Elias D, Contesso G, Genin J, Mankarios H, Rougier P. Tumeurs desmoides ou fibromatoses intra-abdominales [Desmoid tumors or intra-abdominal fibromatoses]. Ann Chir. 1993; 47(4):352–9. English. PMID: 8352514. 8352514

[30] Soufi, M., Lahlou, MK, CHAD, B., BENSAID, M.MESSROURI R. Les tumeurs desmoïdes de la paroi abdominale: à propos de trois cas. RMLG. Medical Review of Liège , 2009 64 (12), 635–640. 20143748

[31] Cavalli M, Bruni PG, Lombardo F, Morlacchi A, Andretto Amodeo C, Campanelli G (2020). Original concepts in anatomy, abdominal-wall surgery, and component separation technique and strategy. Hernia.

[32] Ostruszka P, Ihnát P, Toman D. Transversus abdominis release in the management of a large, chronic defect of the abdominal wall. Rozhl Chir. Spring; 101(5):244–249. English. 2022. 10.33699/PIS.2022.101.5.244-250. (PMID: 35667875). 10.33699/PIS.2022.101.5.244-250 35667875

[33] Dirani M, Chahine E, D'Alessandro A, Chouillard MA, Gumbs AA, Chouillard E (2022). The use of Permacol® biological mesh for complex abdominal wall repair. Minerva Surg.

[34] Benech N, Bonvalot S, Dufresne A, Gangi A, Le Péchoux C, Lopez-Trabada-Ataz D, et al. Thésaurus National de Cancérologie Digestive (TNCD). Desmoid tumors located in the abdomen or associated with adenomatous polyposis: French intergroup clinical practice guidelines for diagnosis, treatment, and follow-up (SNFGE, FFCD, GERCOR, UNICANCER, SFCD, SFED, SFRO, ACHBT, SFR). Dig Liver Dis. 2022:S1590–8658(22)00207–9. 10.1016/j.dld.2022.03.004. PMID: 35508462. 10.1016/j.dld.2022.03.004 35508462

[35] LOUKIL, Issam and ZOUARI, Amine. Tumeur desmoïde géante de la paroi abdominale: à propos d’un cas. Pan African Medical Journal , 2021, vol. 39, No. 1.DOI: 10.11604/pamj.2021.39.211.27965 10.11604/pamj.2021.39.211.27965 PMC8486944 34630823

[36] Garcia-Ortega DY, Martín-Tellez KS, Cuellar- Hubbe M, Martínez-Said H, Álvarez-Cano A, Brener-Chaoul M, Alegría-Baños JA (2020). Martínez - Tlahuel JL. Desmoid-Type Fibromatosis Cancers (Basel).

[37] Toughrai I, Oufkir A, Laalim SA, Majdoub KI, Mazaz K. Recurrent desmoid tumor of the abdominal wall. Pan Afr Med J. 2012; 1:60 p.m. Epub 2012 Nov 20. PMID: 23346274; PMCID: PMC3549445. PMC3549445 23346274

[38] Zhang R, Hu H, Zhang J, Zhang L (2021). Desmoid tumor of the abdominal wall: A case report. Asian J Surg.

[39] Aouati O, Mansoul T, Hassani L (2016). Dermatofibrosarcome de Darier-Ferrand du sein : place de l’imatinib en néoadjuvant. Ann Dermatol Venereol.

[40] Zongo N, Guigemdé RA, Yaméogo PB, Somé RO, Traore B, Dem A (2022). Dermatofibrosarcoma protuberans surgery: experiences of four African surgical oncology units and literature review. J Surg Oncol.

[41] Luther A, Gabriel J, Watson RP, Francis NK (2018). The impact of total body prehabilitation on post-operative outcomes after major abdominal surgery: a systematic review. World J Surg.

[42] Boden I, Skinner EH, Browning L, Reeve J, Anderson L, Hill C, Robertson IK, Story D, Denehy L. Preoperative physiotherapy for the prevention of respiratory complications after upper abdominal surgery: pragmatic, double blinded, multicentre randomised controlled trial. BMJ. 2018 Jan 24;360:j5916. doi: 10.1136/bmj.j5916. Erratum in: BMJ. 2019;365:l1862. PMID: 29367198; PMCID: PMC5782401. 10.1136/bmj.j5916 PMC5782401 29367198

